# Sleep medicine education and knowledge among medical students in selected Saudi Medical Schools

**DOI:** 10.1186/1472-6920-13-133

**Published:** 2013-09-27

**Authors:** Abdulellah Almohaya, Abdulaziz Qrmli, Naeif Almagal, Khaled Alamri, Salman Bahammam, Mashhour Al-Enizi, Atif Alanazi, Aljohara S Almeneessier, Munir M Sharif, Ahmed S BaHammam

**Affiliations:** 1 University Sleep Disorders Center, College of Medicine, King Saud University, Box 225503, Riyadh 11324, Saudi Arabia; 2 College of Medicine, King Saud Bin Abdulaziz University for Health Sciences, Riyadh, Saudi Arabia; 3 Family Medicine Department, College of Medicine, King Saud University, Riyadh, Saudi Arabia; 4 National Plan for Science and Technology, King Saud University, Riyadh, Saudi Arabia

**Keywords:** Sleep medicine, Education, ASKME survey, Medical schools, Medical students, Knowledge

## Abstract

**Background:**

Limited information is available regarding sleep medicine education worldwide. Nevertheless, medical education has been blamed for the under-recognition of sleep disorders among physicians. This study was designed to assess the knowledge of Saudi undergraduate medical students about sleep and sleep disorders and the prevalence of education on sleep medicine in medical schools as well as to identify the obstacles to providing such education.

**Methods:**

We surveyed medical schools that were established more than 10 years ago, asking fourth- and fifth-year medical students (men and women) to participate. Seven medical schools were selected. To assess knowledge on sleep and sleep disorders, we used the Assessment of Sleep Knowledge in Medical Education (ASKME) Survey, which is a validated 30-item questionnaire. The participants were separated into two groups: those who scored ≥60% and those who scored <60%. To assess the number of teaching hours dedicated to sleep medicine in the undergraduate curricula, the organizers of the major courses on sleep disorders were contacted to obtain the curricula for those courses and to determine the obstacles to education.

**Results:**

A total of 348 students completed the survey (54.9% male). Among the participants, 27.7% had a specific interest in sleep medicine. More than 80% of the study sample had rated their knowledge in sleep medicine as below average. Only 4.6% of the respondents correctly answered ≥60% of the questions. There was no difference in the scores of the respondents with regard to university, gender, grade-point average (GPA) or student academic levels. Only five universities provided data on sleep medicine education. The time spent teaching sleep medicine in the surveyed medical schools ranged from 0-8 hours with a mean of 2.6 ±2.6 hours. Identified obstacles included the following: (1) sleep medicine has a lower priority in the curriculum (53%) and (2) time constraints do not allow the incorporation of sleep medicine topics in the curriculum (47%).

**Conclusions:**

Medical students in the surveyed institutions possess poor knowledge regarding sleep medicine, which reflects the weak level of education in this field of medicine. To improve the recognition of sleep disorders among practicing physicians, medical schools must provide adequate sleep medicine education.

## Background

Sleep medicine is a rapidly evolving field of medicine involving diverse types of diseases and affecting nearly all age groups. Sleep disorders are not uncommon and negatively affect morbidity, mortality, quality of life and healthcare utilization [1-3]. The International Classification of Sleep Disorders describes more than 80 different disorders, which can be effectively treated [4]. Obstructive sleep apnea (OSA), which is one of the common sleep disorders affecting 4% of middle-aged males has been linked to several serious medical complications such as hypertension, ischemic heart disease, stroke and insulin resistance [2,3]. Interestingly, most sleep disorders can be treated when diagnosed early. Several studies in different parts of the world have demonstrated clearly that sleep disorders are highly prevalent in all age groups. A National Center on Sleep Disorders study estimated that between 50 and 70 million Americans are affected by a sleep disorder [5]. In Saudi Arabia, several studies have demonstrated that sleep disorders are prevalent among Saudis [6]. Two studies that assessed the prevalence of OSA risk and symptoms among middle-aged Saudi men and women in a primary care setting revealed that three out of ten Saudi men and four out of ten Saudi women are at a high risk of developing OSA [7,8].

Given the magnitude of the problem worldwide, awareness regarding sleep disorders is insufficient among physicians. Thus, a significant number of patients with sleep disorders remain undiagnosed as a result of limited sleep medicine education among healthcare providers [9,10]. One study has demonstrated that the diagnosis of sleep disorders in certain communities is less than 1%, which is considerably lower than the rates indicated by epidemiological studies [11]. A survey of primary healthcare (PHC) physicians in Saudi Arabian primary care centers revealed that only 15% of surveyed physicians have ever attended a lecture on sleep disorders [12]. Another Saudi Arabian study demonstrated that the interval between symptom onset and the diagnosis of narcolepsy was more than 8 years [13]. The authors attributed the delayed diagnosis to the fact that the diagnosis was missed by the treating clinicians in most of the studied patients [13]. Among Saudi women with OSA, other studies have suggested a more than 10-year delay between symptom onset and referral to sleep disorder centers [7,14,15].

Only a limited number of studies have surveyed sleep medicine education in medical schools. An earlier survey in US medical schools revealed that fewer than 2 hours were dedicated to sleep medicine education in medical schools and that 30% of the schools provided no formal sleep medicine education [16]. A more recent survey on sleep education across 12 countries in the Asia-Pacific area and the US/Canada had a poor response; only 25.9% of the surveyed schools completed the survey [17]. Overall, the average time spent on sleep education was less than 2.5 hours. Whereas certain countries provide no education in sleep medicine, developed countries (Australia and the US/Canada) spent approximately 3 hours on such education [17]. In general, most physicians receive no or minimal education on sleep medicine during medical school or residency training [12,18]. No information is available regarding sleep medicine education in Saudi medical schools. Nevertheless, based on published data, it seems that the recognition of sleep disorders among practicing physicians in Saudi Arabia is low [6,12].

Therefore, we conducted this study on Saudi medical schools to examine the following: 1) Sleep medicine knowledge among medical students, 2) The number of hours of teaching dedicated to sleep medicine in different courses in the curriculum related to sleep disorders and 3) The obstacles to sleep medicine education.

## Methods

### Study group

The study was conducted between March 2012 and January 2013. We targeted well-established medical schools in the country that have been established over 10 years. Seven medical schools satisfied this criterion: King Saud University (KSU), Riyadh; King Saud bin Abdulaziz University for Health Sciences (KSAU-HS), Riyadh; King Abdulaziz University (KAU), Jeddah; King Khaled University (KKU), Abha; Umm Al-Qura University (UQU), Makkah; Dammam University (DU), Dammam, and Qassim University (QU), Qassim. A simple random sample of medical students was selected to participate in this study.

### Study protocol

The protocol was approved by the Ethics Committee of our institution, and an informed consent was obtained from all of the participants. Medical education in medical schools in Saudi Arabia comprises 1 year of pre-medical and 5 years of medical school. We targeted medical students attending classes in the fourth (L4) and fifth (L5) academic levels (the final 2 years). This is the period when most clinical postings are completed. The number of students at those two levels was estimated to be 2,853 students. For a confidence interval of ±5 and a confidence level of 95%, the sample size was estimated to be 339 students. A list of students at each university was generated. Then, we selected each sixth student on the list to obtain 480 students to be invited for participation, which was purely voluntarily. The students were assured that the collected data would remain confidential and anonymous. Of the selected group, 348 agreed to participate in the study.

### Survey

Two questionnaires were used. The first targeted undergraduate medical students to assess their knowledge of sleep and sleep disorders, and the second targeted the organizers of the courses related to sleep disorders in six areas (cardiology, otolaryngology, neurology, respiratory medicine, family medicine, physiology and psychiatry) to assess the number of hours assigned to sleep medicine. Because medicine in Saudi Arabia is taught in English, the English language was used in both questionnaires.

### Questionnaire I

The questionnaire for medical students comprised three sections:

1) Demographics, which included age, gender and academic level.

2) Study variables, which included grade-point average (GPA), a specific interest in sleep medicine and a self-evaluation in sleep medicine knowledge.

3) Knowledge assessment: Knowledge in sleep medicine was assessed using the Assessment of Sleep Knowledge in Medical Education (ASKME) Survey, which was designed as a standardized measure for the assessment of medical education in sleep [19]. The ASKME survey is a validated 30-item questionnaire that includes five separate areas of sleep knowledge: 1) basic sleep principles, 2) circadian sleep/wake control, 3) normal sleep architecture, 4) common sleep disorders and 5) the effects of drugs and alcohol on sleep. The items were presented in a “true,” “false” or “I don’t know” format. The questionnaire demonstrated a high degree of internal consistency and reliability among the survey items. The score was determined by adding the correct answers.

Participants were separated into two groups: those who scored ≥60% (the “high score group”) and those who scored <60% (the “low score group”).

### Questionnaire II

The second questionnaire was a short survey adopted from questionnaires used in previous studies [16,17,20]. The questionnaire had two parts. The first section assessed the amount of time assigned to dedicated sleep medicine education in the six selected areas. The second section assessed the obstacles to including sleep medicine topics in the curriculum. The obstacle choices included time constraints, the lack of trained staff/qualified instructors, the lack of resources, lower priority or irrelevance to the program; the course organizer can specify other reasons. Questionnaire II was sent to the organizers of the courses related to sleep disorders in the participating medical schools.

### Statistical analysis

Continuous data are expressed as the mean ± standard deviation (SD), and categorical data are expressed in the text and tables as an absolute number (n) and a percentage (%). The continuous variables were compared using the independent samples *t*-test, and the categorical variables were compared using the chi-square (*χ*^2^) test. A *p* value ≤0.05 was considered to be significant. Standard statistical software (SPSS: Statistical Package for the Social Sciences, v16.0, Chicago, Illinois, USA) was used for the data analysis.

## Results

The participants had a mean age of 23.2 ±1.3 years: 191 students (54.9%) were male, and 217 students (62.4%) were studying in L5. The responses were distributed as follows: KSU = 100 (28.7%), DU = 80 (23%), KAU = 56 (16.1%), QU = 45 (12.9%), KKU = 28 (8%), UQU = 22 (6.3%) and KSAU-HS = 17 (4.9%). Among the participants, 41.5% had a GPA ≥4 out of 5, 48.1% had a GPA of between 3.00-3.99 and 10.4% had a GPA of <3.00. With regard to interest, 27.7% of the sample expressed a specific interest in sleep medicine. When students were asked to rate their knowledge in sleep medicine, they reported the following: excellent: 0%, very good: 2%, average: 16.4%, below average: 40.3% and poor: 41.2%. The mean score of the ASKME questionnaire was 10.39 ±4.44 out of 30. Further analysis showed that the score was <10 in 170 questionnaires (48.9%), 10-20 in 50.6% and >20 in 0.6%. Table 1 presents the percentage of correct answers for each question.

**Table 1:** The percentage and number of correct answers for each question in the ASKME questionnaire given to medical students

**No**	**Questionnaire**	**n (% cpa)**
1	The need for sleep decreases in persons above 50 years of age.	89 (25.6)
2	Melatonin is a natural body hormone that typically increases during nighttime hours.	199 (57.2)
3	More dream sleep (REM) occurs in the second half of the night.	162 (46.6)
4	Sleeping longer on weekends is recommended as a regular practice to make up for loss of sleep during the work week.	180 (51.7)
5	Newborn infants spend approximately 16—18 hours per 24-hour period sleeping.	284 (81.6)
6	The report of insomnia is twice as common in older men than in older women.	93 (26.7)
7	A young (pre-adolescent) child who regularly has trouble getting to sleep at night should be allowed to sleep later in the morning.	174 (50)
8	The typical age of symptom onset for narcolepsy is 40 years or older.	65 (18.7)
9	The ability to sleep increases in persons above 50 years of age.	187 (53.7)
10	Slow-wave sleep is more prominent in the second half of the night.	64 (18.4)
11	The amount of slow-wave sleep increases in persons above 50 years of age.	54 (15.5)
12	Episodes of sleepwalking tend to occur in the last third of the night.	50 (14.4)
13	Episodes of REM sleep tend to lengthen throughout the night.	96 (27.6)
14	Periodic limb movements during sleep are typically decreased in REM sleep.	81 (23.3)
15	Hyperactivity in children can be exacerbated by inadequate sleep.	150 (43.1)
16	In alcoholics in recovery, sleep normalizes within one month of alcohol abstention.	52 (14.9)
17	Daytime napping is recommended for patients with difficulty initiating sleep.	124 (35.6)
18	Weight loss is often indicated in the treatment of primary snoring or mild obstructive sleep apnea.	255 (73.3)
19	Slow-wave sleep is enhanced following daytime exercise.	115 (33)
20	Children who are chronic bedwetters respond to treatment with anticholinergic drugs.	42 (12.1)
21	Nightmares are more common within the first two hours of sleep.	102 (29.3)
22	Heart rate, respiration and blood pressure are more variable during REM sleep compared with non-REM sleep.	167 (48)
23	Antihypertensive drugs (e.g., beta-blockers) may cause sleeping difficulties as a side effect.	88 (25.3)
24	Early morning awakenings in the elderly are often associated with changes in the timing of their biological rhythms.	143 (41.1)
25	Alcohol can be beneficial in reducing the effects of jet lag.	73 (21)
26	Nightshift workers are more likely to fall asleep on the job compared with employees with regular, daytime hours.	203 (58.3)
27	Sleepwalking episodes commonly occur during REM sleep.	50 (14.4)
28	Menopausal women are at higher risk for developing symptoms of sleep apnea compared with pre-menopausal women.	132 (37.9)
29	Irregular sleep scheduling can increase the incidence of sleepwalking in children.	117 (33.6)
30	Symptoms of narcolepsy are related to seizure activity in the brain.	26 (7.5)

**Figure 1:**
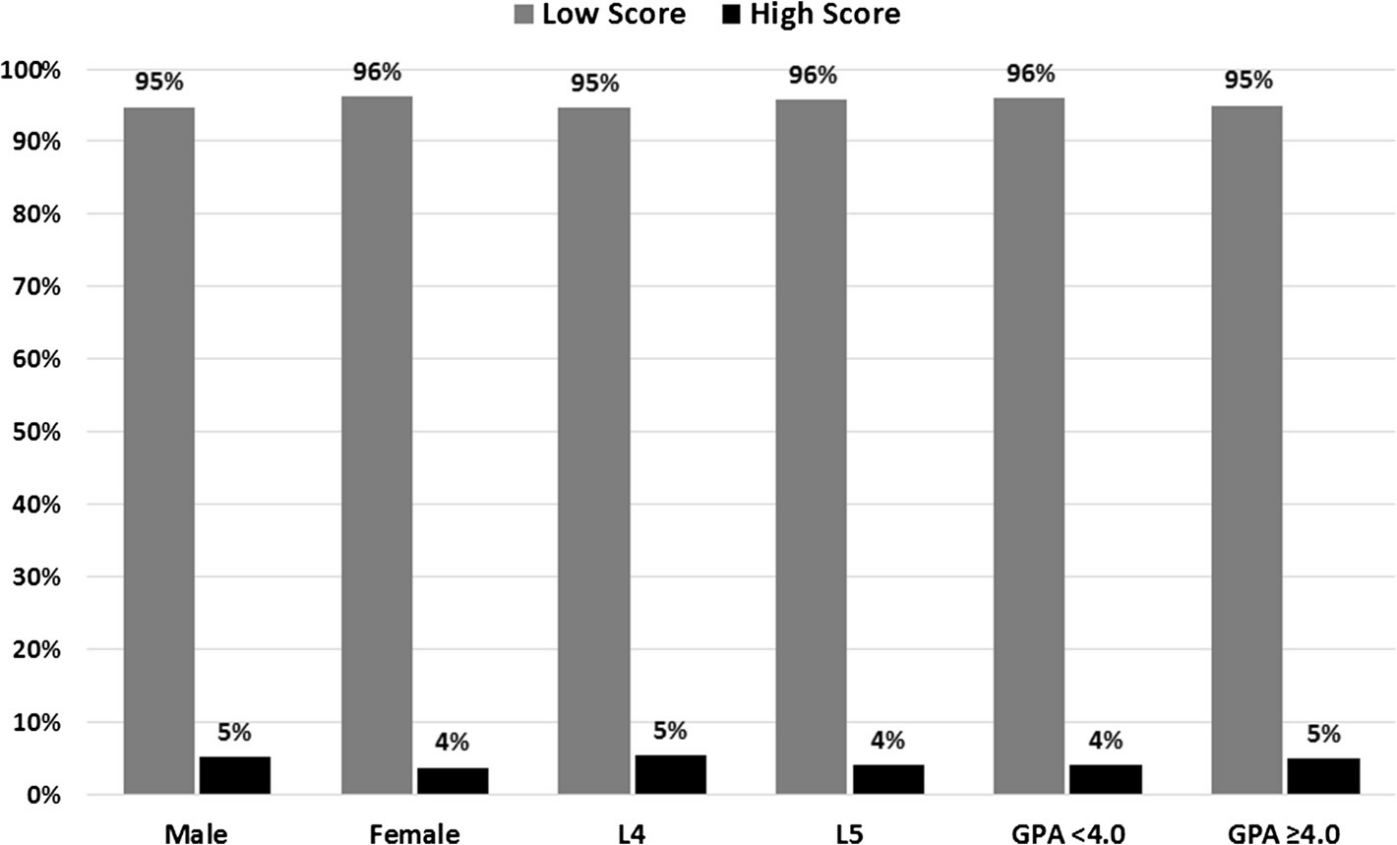
The percentages of high and low scores across sex, academic level and GPA.

**Figure 2:**
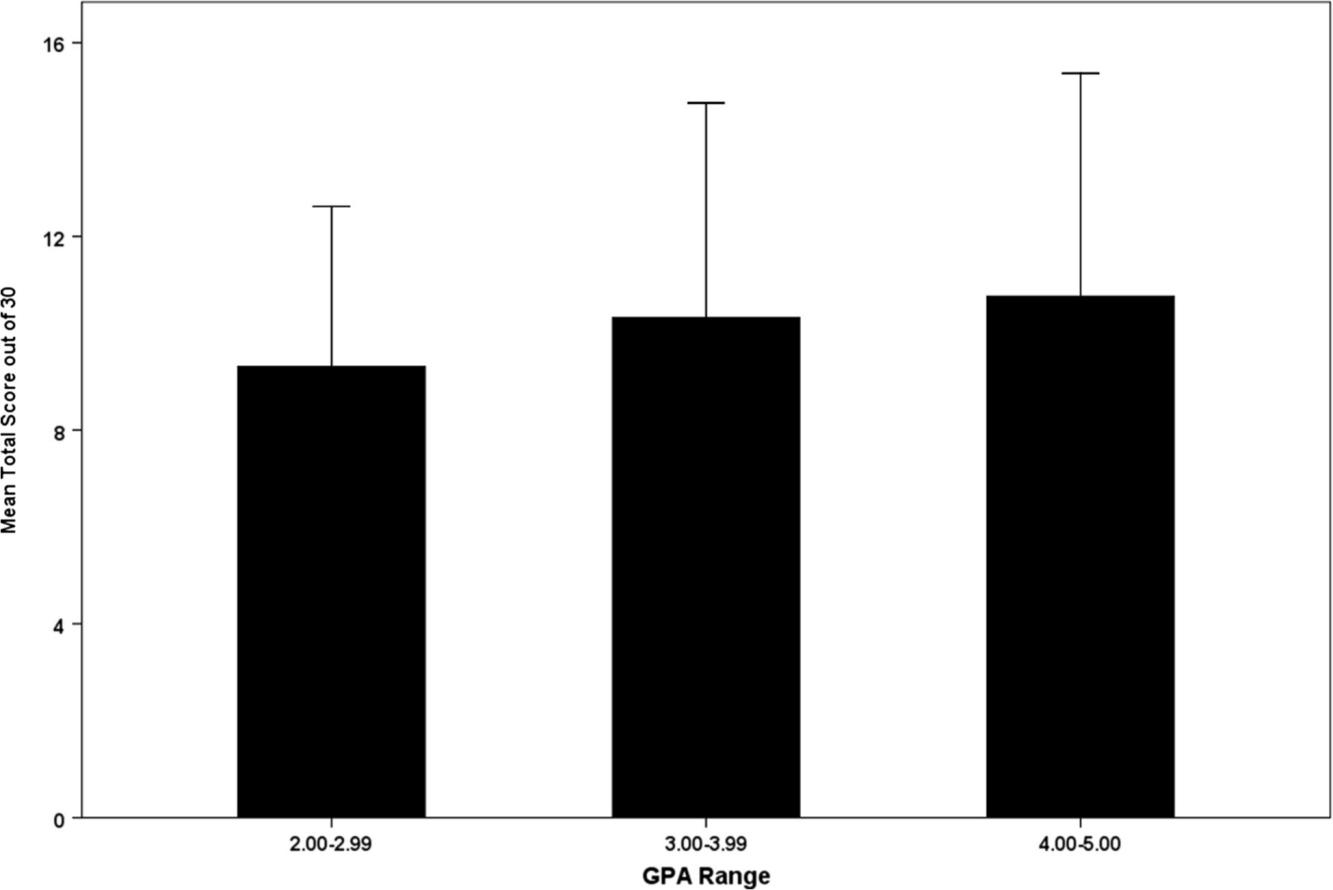
**The mean score in students in three GPA levels (GPA **≥**4, GPA between 3.00-3.99 and GPA** <**3.00).**

Then, the respondents were divided into two groups: those with high scores (≥60%) and those with low scores (<60%). Only 4.6% of the respondents were in the high score group, whereas approximately 95.4% were in the low score group. Figure 1 presents the percentages of high and low scores across sex, academic level and GPA. There was no difference between the high and low score groups with regard to sex, GPA and academic level. There was no difference between the scores of the seven universities. Figure 2 presents the mean score for students in three GPA levels (GPA ≥4, GPA between 3.00-3.99 and GPA <3.00). There was no significant difference between the three levels.

Regarding sleep medicine education, only five universities responded (KSU, KAU, KSUA-HS, DU and KKU). The number of teaching hours dedicated to sleep and sleep disorders in various courses in the responding universities ranged from 0-8 hours with a mean of 2.6 ±2.6 hours. Across all of the medical schools, the medical subcategories with the highest percentage of teaching about sleep and sleep disorders were respiratory medicine (23.1%) and physiology (23.1%), followed by internal medicine (15.4%) and ENT (15.4%), and then family medicine, psychiatry and neurology (each 7.8%).

The identified obstacles to the incorporation of sleep medicine education into the curriculum included the following: (1) sleep medicine has a lower priority in the curriculum (53%) and (2) time constraints do not allow the incorporation of sleep medicine topics (47%).

## Discussion

This study documents that the knowledge of sleep medicine among Saudi medical students is generally low. Moreover, the results show that sleep and sleep disorder education in Saudi medical schools is highly limited despite the substantial increase in the knowledge on and the importance of sleep medicine. Interestingly, there was no difference in the knowledge between academic levels or the different GPA groups, which may indicate that sleep medicine education is sub-optimal in medical schools. It is likely that the students who scored highly acquired their knowledge through self-study [21].

Although several studies have assessed physician knowledge regarding OSA [12,22-25], the number of studies that assessed sleep medicine knowledge among medical students is limited. In the present study, only 4.6% of the respondents correctly answered ≥ 60% of the questions. Previous studies reported similar data for different countries. In a study that assessed the knowledge of sleep medicine among medical students in Singapore, Manhendran and Chan reported that sleep medicine knowledge was low among medical students, with 46.7% scoring between 1 and 10 out of 30, 51.7% scoring between 11-20 out of 30 and only 1.7% scoring between 21 and 30 points [21]. In another study from Croatia that used the same questionnaire, Kovacic et al. reported that the proportion of correct answers among medical students was 0.41 [26].

A recent study in China using a different assessment questionnaire revealed that Chinese medical students knew little about sleep disorders [27]. Similar results were reported among practicing physicians. A US survey demonstrated that primary care physicians do not realize the health impact of sleep disorders [11]. Another study revealed that PHC physicians rated their knowledge of sleep medicine as fair or poor [25]. A survey of PHC physicians in Saudi Arabia revealed that PHC physicians do not completely recognize the importance and impact of OSA and other sleep disorders [12]. Whereas 43% of the participants did not realize the existence of sleep medicine as a specialty, 40% felt that sleep disorders are not common [12].

The lack of knowledge regarding sleep medicine and sleep disorders among medical students is the result of the limited time assigned for teaching sleep medicine in medical schools. In addition, Teodorescu et al. reported that sleep medicine was represented in <2% of the content in 31 textbooks on four major specialties (neurology, psychiatry, pulmonary medicine and geriatrics) [28]. During 5 years of medical education in Saudi medical schools, fewer than 3 hours were allocated to teaching sleep medicine. Only five medical schools responded. This lack of response may reflect the busy schedule of the course organizers. Nonetheless, it is possible that the non-responding schools may not want to report the “limited time” or “no time” dedicated for sleep education in their schools. It is likely that the reported results overestimate the time dedicated to sleep education because the schools that did not respond were possibly less likely to have dedicated teaching time to sleep education.

A 1993 survey of medical schools in the US reported that fewer than 2 hours of the medical education curriculum were devoted to sleep and sleep disorders [16]. A more recent survey on sleep education in the medical school curriculum in 12 countries in the Asia-Pacific region and North America reported that the overall time allocated to sleep education was less than 2.5 hours [17]. Moreover, 27% of the responding schools reported that their schools provide no sleep education [17]. The US/Canada and Australia were the only countries that provided more than 3 hours of sleep education.

In the present study, the two identified obstacles to increasing the time allocated for sleep medicine education were as follows: 1) the topic’s low priority in the curriculum (53%) and 2) insufficient time (47%). In a survey by Mindell et al. across 12 countries, the following obstacles were identified: insufficient time (32%), a lack of qualified staff (24%), a lack of resources (17%), low priority (17%) and irrelevance (7%). It is of concern that despite the increased knowledge regarding the high prevalence of sleep disorders and their impact on health, 53% of the surveyed course organizers believe that sleep medicine education is a low priority for medical students.

Intuitively, the early detection and management of patients with sleep disorders depends considerably on the knowledge and awareness of practicing physicians. Because sleep medicine education in medical schools is highly limited, it is likely that sleep disorders will be under-recognized and that patients with these disorders may be inaccurately diagnosed and may receive inappropriate treatment. It has been demonstrated that doctors who receive training in sleep disorders are more likely to recognize sleep disorders [11].

A major challenge for the future is encouraging the educational system at all levels to acknowledge the high prevalence and serious consequences of sleep disorders. The curricula of modern medical schools must address a substantial amount of scientific and clinical material in a limited time. Therefore, it is difficult to devote a block for sleep and sleep disorder education. Harding et al. proposed alternative methods to integrate sleep topics into existing curriculum blocks [29]. The suggested approaches included integrating basic sleep science topics into the problem-based curricula of the preclinical years (such as physiology, neuroanatomy and neuroscience), integrating sleep history and physical signs into introductory clinical medicine and integrating sleep disorders into problem-solving sessions. These same researchers proposed the following measures for the clinical years: including sleep topics in the clinical rotations (such as internal medicine, psychiatry, family medicine and neurology), using computer-based simulations for different sleep disorders [30] and allowing students to take elective courses in sleep medicine clinics [29]. In addition, it is important to cover sleep-related material in qualifying exams, which will encourage the inclusion of sleep medicine in medical education and competency-based learning [17]. The above can be implemented in Saudi medical schools as most medical schools use the problem-based learning strategy.

## Conclusion

In conclusion, Saudi medical students have poor knowledge of sleep medicine, which reflects the weak level of education in this field of medicine. Normal sleep is an essential component of human health and well-being. Therefore, it is essential to integrate sleep and sleep disorders into medical school education. It is hoped that medical schools will provide adequate education in sleep medicine in the near future.

## Abbreviations

OSA: Obstructive sleep apnea; PHC: Primary healthcare; US: United States; L4: Fourth academic level; L5: Fifth academic level; ASKME: Assessment of sleep knowledge in medical education; KSU: King Saud University; KSAU-HS: King Saud bin Abdulaziz for health sciences; KAU: King Abdulaziz University; KKU: King Khaled University; UQU: Umm Al-Qura University; DU: Dammam University; QU: Qassim University; GPA: Grade point average.

## Competing interests

The authors declare that that they have no competing interests.

## Authors’ contributions

AA: conception, design and data collection, manuscript writing. AQ, NA, KA, SB, MA and AA: conception, design and data collection. ASA: Conception, data analysis and manuscript writing. MS: design, data analysis and manuscript writing. AB: conception, design, data analysis, manuscript writing and approval and securing funding. All authors read and approved the final manuscript.

## Authors’ information

AA and AQ: Medical intern. NA, KA, SB, MA and AA: Medical student. ASA: Medical education specialist. MS: data manager and statistician. AB: sleep medicine specialist.

## Pre-publication history

The pre-publication history for this paper can be accessed here:

http://www.biomedcentral.com/1472-6920/13/133/prepub

## References

[B1] Skaer TL Sclar DA Economic implications of sleep disorders Pharmacoeconomics 2010 28 1015 1023 10.2165/11537390-000000000-00000 20936885

[B2] Hossain JL Shapiro CM The prevalence, cost implications, and management of sleep disorders: an overview Sleep Breath 2002 6 85 102 10.1055/s-2002-32322 12075483

[B3] Young T Finn L Peppard PE Szklo-Coxe M Austin D Nieto FJ Stubbs R Hla KM Sleep disordered breathing and mortality: eighteen-year follow-up of the Wisconsin sleep cohort Sleep 2008 31 1071 1078 18714778 PMC2542952

[B4] American Academy of Sleep Medicine International classification of sleep disorders: Diagnostic and coding manual 2005 2 Westchester. IL: American Academy of Sleep Medicine

[B5] 2003 National Sleep Disorders Research Plan Sleep 2003 26 253 257 12749542

[B6] Bahammam AS Sleep medicine in Saudi Arabia: Current problems and future challenges Ann Thorac Med 2011 6 3 10 10.4103/1817-1737.74269 21264164 PMC3023868

[B7] Bahammam AS Al-Rajeh MS Al-Ibrahim FS Arafah MA Sharif MM Prevalence of symptoms and risk of sleep apnea in middle-aged Saudi women in primary care Saudi Med J 2009 30 1572 1576 19936423

[B8] BaHammam AS Alrajeh MS Al-Jahdali HH BinSaeed AA Prevalence of symptoms and risk of sleep apnea in middle-aged Saudi males in primary care Saudi Med J 2008 29 423 426 18327372

[B9] Young T Palta M Dempsey J Skatrud J Weber S Badr S The occurrence of sleep-disordered breathing among middle-aged adults N Engl J Med 1993 328 1230 1235 10.1056/NEJM199304293281704 8464434

[B10] Stores G Cappuccio FP, Miller MA, Lockley SW “Misdiagnosis of sleep disorders in adults and children: implications for clinical practice and epidemiology” Sleep, Health and Society: From Aetiology to Public Health 2011 1: Oxford University Press

[B11] Rosen RC Zozula R Jahn EG Carson JL Low rates of recognition of sleep disorders in primary care: comparison of a community-based versus clinical academic setting Sleep Med 2001 2 47 55 10.1016/S1389-9457(00)00043-5 11152982

[B12] BaHammam AS Knowledge and attitude of primary health care physicians towards sleep disorders Saudi Med J 2000 21 1164 1167 11360092

[B13] BaHammam AS Alenezi AM Narcolepsy in Saudi Arabia. Demographic and clinical perspective of an under-recognized disorder Saudi Med J 2006 27 1352 1357 16951772

[B14] Alotair H Bahammam A Gender differences in Saudi patients with obstructive sleep apnea Sleep Breath 2008 12 323 329 10.1007/s11325-008-0184-8 18369671

[B15] Bahammam AS Sleep medicine: Present and future Ann Thorac Med 2012 7 3 113 114 10.4103/1817-1737.98841 22924066 PMC3425040

[B16] Rosen RC Rosekind M Rosevear C Cole WE Dement WC Physician education in sleep and sleep disorders: a national survey of U.S. medical schools Sleep 1993 16 249 254 8506458 10.1093/sleep/16.3.249

[B17] Mindell JA Bartle A Wahab NA Ahn Y Ramamurthy MB Huong HT Kohyama J Ruangdaraganon N Sekartini R Teng A Goh DY Sleep education in medical school curriculum: a glimpse across countries Sleep Med 2011 12 928 931 10.1016/j.sleep.2011.07.001 21924951

[B18] Rosen R Zozula R Education and training in the field of sleep medicine Curr Opin Pulm Med 2000 6 512 518 10.1097/00063198-200011000-00009 11100962

[B19] Zozula R Bodow M Yatcilla D Cody R Rosen RC Development of a brief, self-administered instrument for assessing sleep knowledge in medical education: “the ASKME Survey” Sleep 2001 24 227 233 11247060

[B20] Mindell JA Bartle A Ahn Y Ramamurthy MB Huong HT Kohyama J Li AM Ruangdaraganon N Sekartini R Teng A Sleep education in pediatric residency programs: a cross-cultural look BMC Res Notes 2013 6 130 10.1186/1756-0500-6-130 23552445 PMC3621514

[B21] Mahendran R Subramaniam M Chan YH Medical students’ behaviour, attitudes and knowledge of sleep medicine Singapore Med J 2004 45 587 589 15568121

[B22] Wang CL Li XZ Cai XL Pan XL Min J Anesthesiologist’s knowledge and attitudes about obstructive sleep apnea: a survey study Sleep Breath 2012 16 41 46 10.1007/s11325-011-0482-4 21240655

[B23] Southwell C Moallem M Auckley D Cardiologist’s knowledge and attitudes about obstructive sleep apnea: a survey study Sleep Breath 2008 12 295 302 10.1007/s11325-008-0170-1 18327624

[B24] Bian H Knowledge, opinions, and clinical experience of general practice dentists toward obstructive sleep apnea and oral appliances Sleep Breath 2004 8 85 90 10.1055/s-2004-829633 15211392

[B25] Papp KK Penrod CE Strohl KP Knowledge and attitudes of primary care physicians toward sleep and sleep disorders Sleep Breath 2002 6 103 109 10.1055/s-2002-34317 12244489

[B26] Kovacic Z Marendic M Soljic M Pecotic R Kardum G Dogas Z Knowledge and attitude regarding sleep medicine of medical students and physicians in Split, Croatia Croat Med J 2002 43 71 74 11828565

[B27] Luo M Feng Y Li T Sleep medicine knowledge, attitudes, and practices among medical students in Guangzhou, China Sleep Breath 2013 17 687 693 10.1007/s11325-012-0743-x 22752711

[B28] Teodorescu MC Avidan AY Teodorescu M Harrington JJ Artar AO Davies CR Chervin RD Sleep medicine content of major medical textbooks continues to be underrepresented Sleep Med 2007 8 271 276 10.1016/j.sleep.2006.09.001 17369089

[B29] Harding SM Berner ES Developing an action plan for integrating sleep topics into the medical school curriculum Sleep Breath 2002 6 155 160 10.1055/s-2002-36526 12524568

[B30] Quan SF Anderson JL Hodge GK Use of a supplementary internet based education program improves sleep literacy in college psychology students J Clin Sleep Med 2013 9 155 160 23372469 10.5664/jcsm.2414 PMC3544384

